# A stacking ensemble model for predicting the occurrence of carotid atherosclerosis

**DOI:** 10.3389/fendo.2024.1390352

**Published:** 2024-07-23

**Authors:** Xiaoshuai Zhang, Chuanping Tang, Shuohuan Wang, Wei Liu, Wangxuan Yang, Di Wang, Qinghuan Wang, Fang Tang

**Affiliations:** ^1^ Department of Data Science, School of Statistics and Mathematics, Shandong University of Finance and Economics, Jinan, China; ^2^ Information Technology Division, Shandong International Trust Co., Ltd., Jinan, China; ^3^ Department of Medical Ultrasound, The First Affiliated Hospital of Shandong First Medical University & Shandong Provincial Qianfoshan Hospital, Shandong Engineering Research Center of Diagnosis and Treatment Technology for Bariatric and Metabolism-Associated Surgery, Jinan, China; ^4^ School of Public Health, Harbin Medical University, Harbin, China; ^5^ Center for Big Data Research in Health and Medicine, The First Affiliated Hospital of Shandong First Medical University & Shandong Provincial Qianfoshan Hospital Shandong Data Open Innovative Application Laboratory, Jinan, China; ^6^ Shandong Provincial Qianfoshan Hospital, Cheeloo College of Medicine, Shandong University, Jinan, China

**Keywords:** carotid atherosclerosis, endocrine-related markers, prediction, stacking, machine learning

## Abstract

**Background:**

Carotid atherosclerosis (CAS) is a significant risk factor for cardio-cerebrovascular events. The objective of this study is to employ stacking ensemble machine learning techniques to enhance the prediction of CAS occurrence, incorporating a wide range of predictors, including endocrine-related markers.

**Methods:**

Based on data from a routine health check-up cohort, five individual prediction models for CAS were established based on logistic regression (LR), random forest (RF), support vector machine (SVM), extreme gradient boosting (XGBoost) and gradient boosting decision tree (GBDT) methods. Then, a stacking ensemble algorithm was used to integrate the base models to improve the prediction ability and address overfitting problems. Finally, the SHAP value method was applied for an in-depth analysis of variable importance at both the overall and individual levels, with a focus on elucidating the impact of endocrine-related variables.

**Results:**

A total of 441 of the 1669 subjects in the cohort were finally diagnosed with CAS. Seventeen variables were selected as predictors. The ensemble model outperformed the individual models, with AUCs of 0.893 in the testing set and 0.861 in the validation set. The ensemble model has the optimal accuracy, precision, recall and F1 score in the validation set, with considerable performance in the testing set. Carotid stenosis and age emerged as the most significant predictors, alongside notable contributions from endocrine-related factors.

**Conclusion:**

The ensemble model shows enhanced accuracy and generalizability in predicting CAS risk, underscoring its utility in identifying individuals at high risk. This approach integrates a comprehensive analysis of predictors, including endocrine markers, affirming the critical role of endocrine dysfunctions in CAS development. It represents a promising tool in identifying high-risk individuals for the prevention of CAS and cardio-cerebrovascular diseases.

## 1 Introduction

Carotid atherosclerosis (CAS) is a multifaceted disease characterized by the progressive accumulation of atherosclerotic plaques within the carotid arteries (1). As a manifestation of atherosclerosis in local blood vessels, the continuous development of CAS is a major and potentially preventable cause of ischaemic stroke (2). Early manifestations of CAS such as intermittent dizziness or mild headaches are subtle, often leading to missed diagnoses. As CAS progresses, it severely impacts the physical and psychological well-being of individuals, imposing substantial financial strains on their families. Therefore, early prediction and prevention of CAS are crucial to mitigate the risk of subsequent cardio-cerebrovascular events.

Current research on CAS has mainly focused on the analysis of risk factors, the most common of which include age, smoking status, physical inactivity, abnormal blood glucose levels, hypertension and others (3–7). These factors, particularly hyperglycemia and hypertension, indicative of underlying metabolic and hormonal imbalances, contribute to the endothelial dysfunction, inflammation, and subsequent plaque formation characteristic of atherosclerosis. Despite numerous studies on CAS risk factors, there is a scarcity of research dedicated to developing predictive models for CAS, with existing models primarily using cross-sectional data for disease diagnosis rather than predicting its onset.

Machine learning methods offer the potential to achieve precise predictive ability to assess diagnostic and prognostic outcomes (8–11). Among various machine learning approaches, ensemble learning, which includes techniques like bagging (e.g., random forests), boosting (e.g., XGBoost, GBDT), and stacking, stands out by integrating multiple weak classifiers to form a robust classifier, thereby improving prediction accuracy and model generalizability (12–15). Stacking ensemble models, which train different weak learners in parallel, have shown superior performance across various domains, from healthcare to financial forecasting (16–20). However, they also present significant challenges such as computational complexity and a lack of interpretability, often referred to as the “black box” phenomenon, which can obscure understanding of decision-making processes (21).

In this study, we employ a stacking ensemble learning algorithm to construct a risk prediction model for the occurrence of CAS based on a routine health checkup cohort. The predictive performance of the ensemble model was compared with that of the individual models. We utilize the SHapley Additive exPlanation (SHAP) method (22) to elucidate the predictive relationships between CAS and various risk factors, with a particular focus on endocrine-related markers.

## 2 Materials and methods

### 2.1 Study design and data collection

The study cohort was derived from the routine health check-up system of the First Affiliated Hospital of Shandong First Medical University in Jinan, China. All the participants were free of CAS at the first check-up and underwent three health checks during the follow-up. Individuals who had been diagnosed with coronary heart disease, previous coronary heart disease, cerebral ischaemia, cerebral infarction, cerebral artery stenosis, cerebral artery spasm, coronary artery stenosis, coronary atherosclerotic heart disease, and those with missing information were excluded. CAS was diagnosed by carotid B-mode ultrasonography as a carotid intima-media thickness of 1.0 mm or greater or plaque formation. The study was approved by the Ethics Committee of the First Affiliated Hospital of Shandong First Medical University, and informed consent was obtained from all eligible participants.

### 2.2 Study variables

The study variables consisted of three sets of data: demographic data, laboratory indicators, and clinical history. All the individuals in this study cohort underwent anthropometric and laboratory tests. The height and weight of the participants were measured while they were wearing light clothing and no shoes. Peripheral blood samples were collected from the subjects after an overnight fast, and the variables included blood urea nitrogen (BUN), lymphocyte percentage (LYM), aspartate aminotransferase (AST), red cell volume distribution width standard deviation (RDW-SD), red blood cell count (RBC), mean corpuscular haemoglobin concentration (MCHC), mean platelets (MPV), fasting blood glucose (GLU), platelet count (PLT), eosinophil percent (PEOS), white blood cell count (WBC), and carcinoembryonic antigen (CEA). Disease history was also collected, such as carotid stenosis (CS), diabetes mellitus (DM), and hypertension. All the measurements were collected following the same standard procedures.

### 2.3 SMOTE sampling

To address the issue of data imbalance, we applied the synthetic minority oversampling technique (SMOTE) in our study. Since the number of individuals without CAS was larger than those with CAS, SMOTE was employed to generate synthetic samples of the minority class (22). The new synthetic records were generated using the existing samples of the minority class via linear interpolation. After we obtain new minority sample data, a balanced dataset can be obtained by merging with majority samples.

### 2.4 Variable selection

Variable selection is an important step in the application of machine learning to ensure that the most relevant predictors are used. Both filtering and embedded feature selection methods were used to select the predictors. The variables were first selected using univariate logical regression with a threshold *P* value of 0.1. Second, we applied three tree-based machine learning methods-random forest (RF), eXtreme Gradient Boost (XGBoost), and gradient boosting decision tree (GBDT))-to assess the importance of each variable. These methods are well-suited for identifying important variables due to their ability to capture complex interactions and non-linear relationships. Variables were ranked based on their importance scores from these models. Finally, the correlation coefficients of the continuous variables were calculated to address multicollinearity, which can distort the model’s estimates and reduce interpretability. Features with low importance among the highly related variables were eliminated for determining the predictors.

### 2.5 Statistical analysis

**Figure 1:**
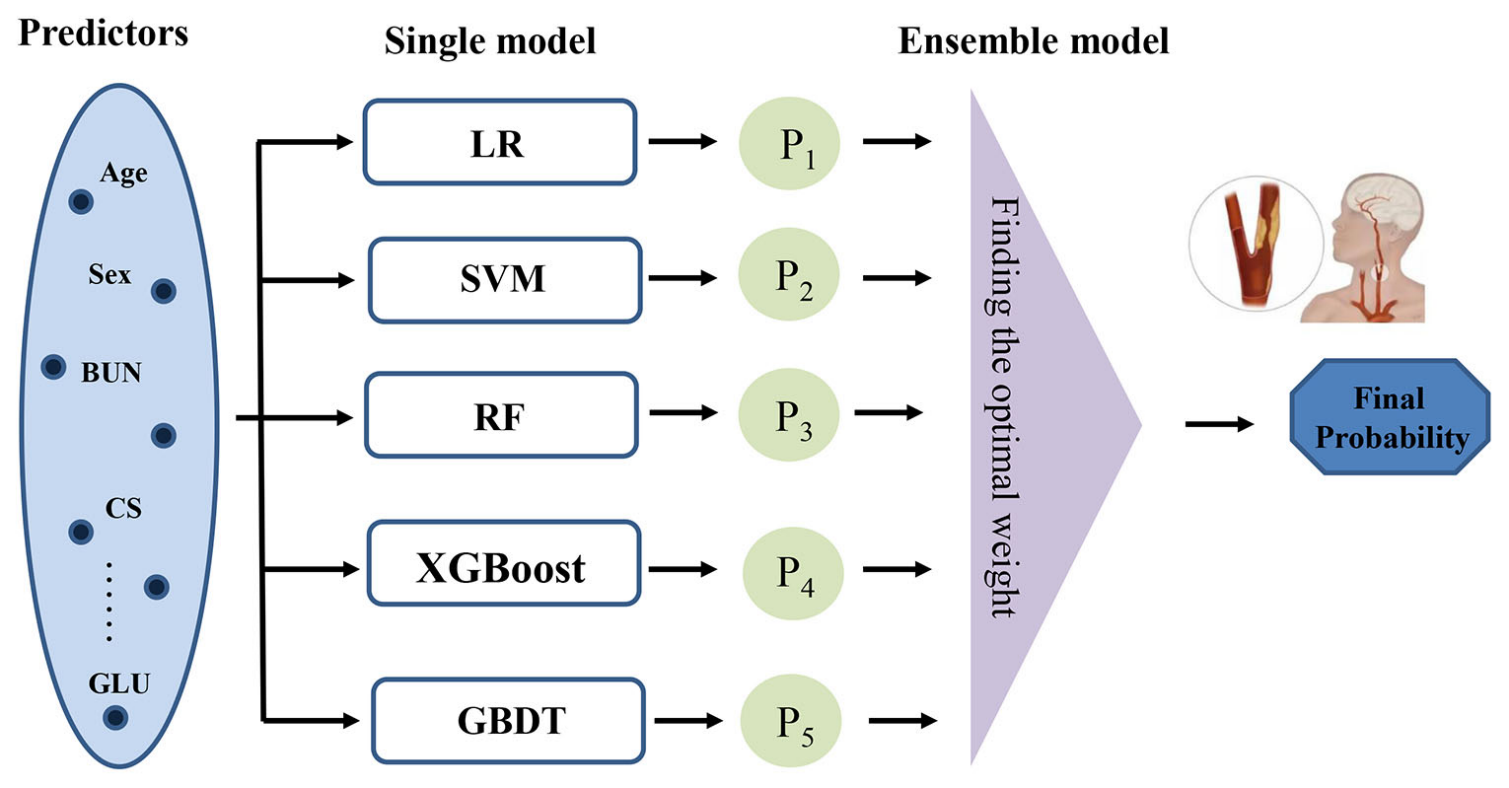
Framework of the stacking ensemble model. (LR, logistic regression; RF, random forest; SVM, support vector machine; XGBoost, extreme gradient boosting; GBDT, gradient boosting decision tree.

The baseline characteristics were assessed for CAS and non-CAS patients during the follow-up. Continuous variables were described by the mean and standard deviation (SD), and categorical features were described as proportions; we compared the baseline features using the *t* test and the chi-square test. To predict the probability of CAS, we employed five machine learning models: logistic regression (LR), support vector machine (SVM), RF, XGBoost, and GBDT (11, 23–26). We then used a stacking ensemble model, specifically the super learner, which combines these individual models by assigning them different weights to optimize predictive performance (27). The final predicted value is a weighted sum of the individual model predictions, where the weights are determined to minimize the cross-validation risk (28). **Figure 1** shows the framework of the super learner ensemble model. We compared the predictive performance of the super learner against the individual models (LR, RF, SVM, XGBoost, and GBDT).

### 2.6 Hyperparameter tuning

We optimized the hyperparameters using a random search method, which is a traditional and efficient technique for tuning in classification methods (29).This process was conducted within a 5-fold cross-validation framework. Specifically, each model configuration was trained on four folds and validated on the remaining fold. This cycle was repeated five times, with each fold serving as the validation set once, to ensure a comprehensive evaluation across the entire dataset. We performed the random search 1,000 times, selecting the hyperparameter combination with the highest average areas under the receiver operating characteristic curve (AUC).

### 2.7 Predictive performance assessment

The performance of the prediction model was validated using both the testing and hold-out validation set. Several metrics were used to evaluate the performance of the prediction models: accuracy, precision, recall, F1 score, and AUC. Compared to commonly used performance metrics, the AUC better reflects model performance in unbalanced datasets. Hence, the AUC was the main metric, while the others were considered of secondary priority.

### 2.8 Model interpretation

To solve the “black box” problem in machine learning, we report the feature importance ranking of each predictor based on SHAP values (22). SHAP values are useful for explaining the prediction of a machine learning model by computing the contribution of each feature to the prediction. Kernel-based SHAP values were used to rank the variables in terms of their ability to predict the CAS, which is an additive feature attribution method using kernel functions enabling consistent explanation of feature importance (30).

## 3 Results

### 3.1 Data description

**Figure 2:**
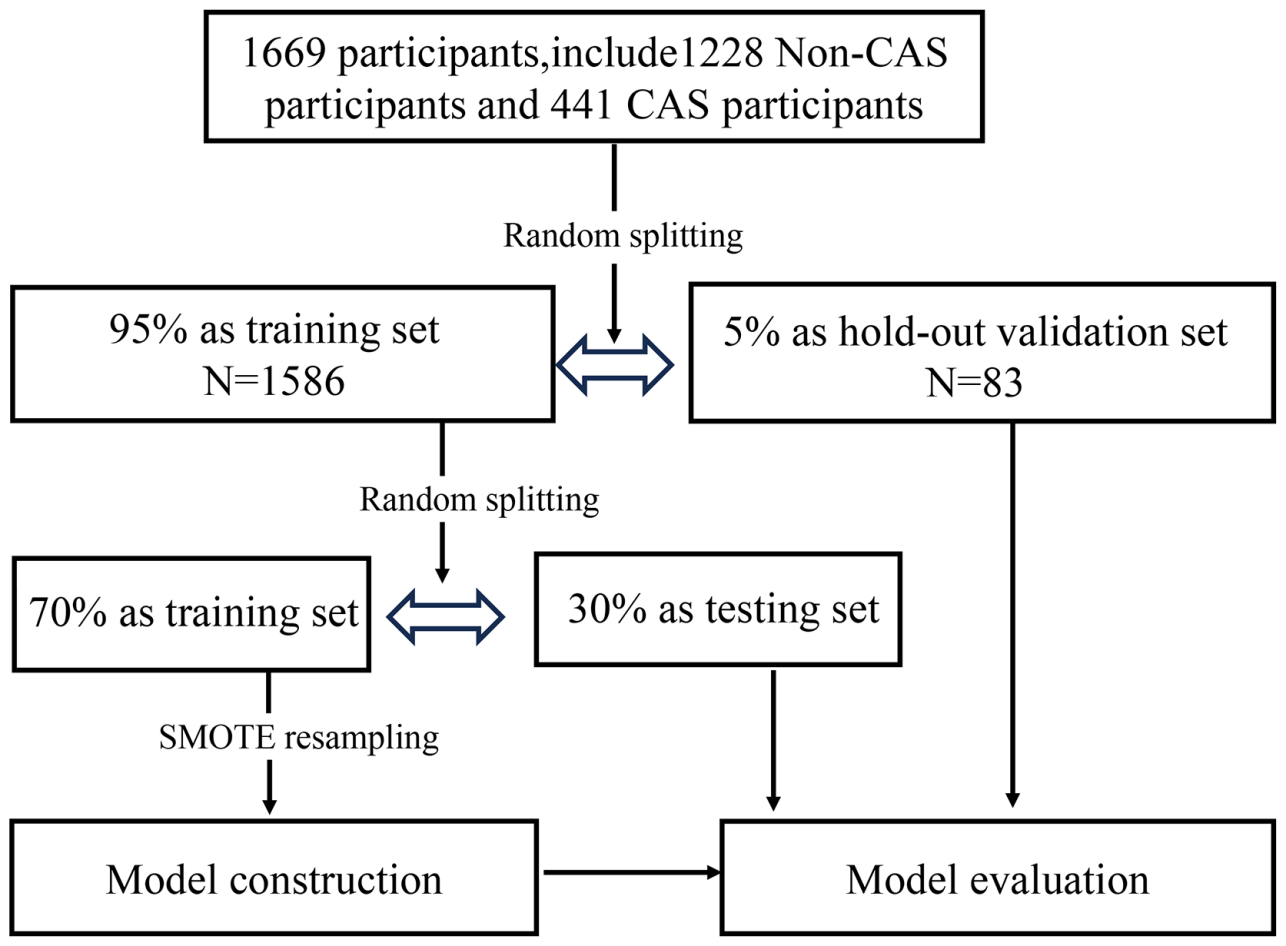
Dataset partitioning in the modelling process.

A total of 1669 participants were included in this study, including 1426 (85.4%) males and 243 (14.6%) females. A total of 441 participants were diagnosed with CAS during the follow-up, including 395 men and 46 women. A total of 1228 participants were not diagnosed with CAS. The SMOTE method was used to address the sample imbalance problem. **Figure 2** shows the roadmap of the data processing. All the samples were first divided into a training set and a hold-out validation set. Specifically, 5% of the subjects were randomly selected in advance as the validation set, and the remaining 95% were used for model construction; 419 patients with CAS and 1167 non-CAS patients were included. Of the remaining data, 70% of the data were used as the training set where SMOTE resampling was applied to address class imbalance. The remaining 30% was used as the testing set. In the original dataset, the ratio of non-CAS to CAS was approximately 2.78 to 1, which was adjusted in the training set to approximately 1 to 1.

### 3.2 Variable selection results

A total of 28 variables with a *P* value < 0.1 were retained in the univariate logistic regression and subsequently included in the three machine learning models. **Supplementary Figure S1** shows the variable importance rankings for RF, GBDT, and XGBoost. A total of 22 of these variables were common among all three machine learning algorithms (please see the **Supplementary File**). The correlations between the selected continuous variables are shown in **Supplementary Figure S2** in the **Supplementary File**. Variables with high correlations and the lowest importance were removed; thus, five variables were excluded. Finally, 17 variables were selected, including age, sex, BUN, LYM, AST, RDW-SD, RBC, MCHC, MPV, GLU, PLT, PEOS, WBC, CEA, CS, hypertension and DM.

**Table 1** summarizes the baseline characteristics of the incident CAS status. Overall, individuals who developed CAS were more likely to be male CS, DM, hypertension, older age, MCHC, BUN, RDW-SD, MPV, GLU, PEOS, WBC, and CEA and lower PLT, LYM, AST, and RBC at baseline; these variables were significantly different at a level of 0.1.

**Table 1:** Baseline characteristics by incident CAS status.

Variables	N=1669	Non-CAS N=1228 (73.6%)	CAS N=441 (26.4%)	*t/X* ^2^	*P*
Age	55.1 ± 7.6	53.7 ± 6.2	59.1 ± 9.4	-11.28	<0.001
MCHC	339.4 ± 10.1	339.0 ± 10.4	340.3 ± 9.3	-2.29	0.022
PLT	221.1 ± 50.9	222.6 ± 50.2	216.9 ± 52.7	1.98	0.048
BUN	5.2 ± 1.3	5.2 ± 1.3	5.4 ± 1.3	-2.39	0.017
LYM	0.4 ± 0.1	0.4 ± 0.1	0.3 ± 0.1	3.29	0.001
AST	20.0 ± 8.0	20.4 ± 8.5	19.0 ± 6.0	3.72	<0.001
RDW-SD	42.4 ± 2.7	42.2 ± 2.7	42.8 ± 2.6	-3.63	<0.001
RBC	4.9 ± 0.4	4.9 ± 0.4	4.8 ± 0.4	2.46	0.014
MPV	10.3 ± 0.9	10.3 ± 0.9	10.4 ± 0.8	-2.52	0.012
GLU	5.7 ± 1.3	5.6 ± 1.1	5.9 ± 1.7	-4.43	<0.001
EOS	0.026 ± 0.022	0.025 ± 0.020	0.028 ± 0.026	-1.87	0.061
WBC	6.3 ± 1.6	6.2 ± 1.5	6.4 ± 1.7	-2.29	0.023
CEA	2.0 ± 1.4	1.9 ± 1.3	2.1 ± 1.6	-2.93	0.004
Sex: n (%) 0 1	1426(85.4%) 243(14.6%)	1031(84.0%) 197(16.0%)	395(89.6%) 46 (10.4%)	8.21	0.004
CS: n (%) 0 1	1088(65.2%) 581 (34.8%)	895(72.9%) 333(27.1%)	193(43.8%) 248(56.2%)	121.24	<0.001
DM: n (%) 0 1	1475(88.4%) 194(11.6%)	1115(90.8%) 113(9.2%)	360(81.6%) 81(18.4%)	26.53	<0.001
Hypertension: n (%) 0 1	1150(68.9%) 519(31.1%)	895(72.9%) 333(27.1%)	255(57.8%) 186(42.2%)	34.34	<0.001

### 3.3 Model comparison

The super learner algorithm creates an optimal weighted average of the five models (LR, RF, SVM, XGBoost, and GBDT). **Supplementary Table S1** in the **Supplementary File** depicts the weight coefficients of the super learner model. **Supplementary Table S2** shows the hyperparameters used in the models. The weight coefficients of LR and SVM are 0, indicating that they were not used in the super learner model, while the coefficient of RF is 0.820, which is much greater than that of the other four models, indicating that RF contributes most in the prediction model.

**Table 2** shows the predictive performance of the six models on the testing set and the hold-out validation set. It can be seen that the predictive performance varies across the five models. SVM has the highest precision, while its performance in the validation set is inferior. Logistic regression had the lowest performance in the testing set. These results also indicate the reason that the two methods are not selected in the super learner model. Combined with the advantages of RF, XGBoost and GBDT, the performance of the super learner was improved. The super learner model had the optimal performance measures in the validation set, with considerable performance in the testing set. The ROC curves of the different machine learning models are shown in **Supplementary Figure S3**. The super learner has the largest ROC curve area in the validation set, and its AUC is 0.861. Overall, the predictive performance of the super learner model is superior to that of the other five models, especially regarding the overfitting problem.

**Table 2:** Predictive performance of the six machine learning models.

Models	Performance metrics^a^				
	accuracy	precision	recall	F1	AUC
LR	0.734(0.783)	0.732(0.563)	0.701(0.818)	0.716(0.667)	0.797(0.857)
SVM	0.825(0.602)	0.793(0.359)	0.859(0.636)	0.825(0.459)	0.909(0.639)
RF	0.805(0.759)	0.783(0.528)	0.822(0.864)	0.802(0.655)	0.891(0.848)
XGBoost	0.777(0.723)	0.761(0.487)	0.780(0.864)	0.770(0.623)	0.872(0.817)
GBDT	0.789(0.759)	0.771(0.529)	0.797(0.818)	0.784(0.642)	0.866(0.828)
Super learner	0.795(0.795)	0.785(0.571)	0.788(0.909)	0.786(0.701)	0.893(0.861)

a The predictive performance values for the hold-out validation set are shown in parentheses.

### 3.4 Model interpretation

**Figure 3:**
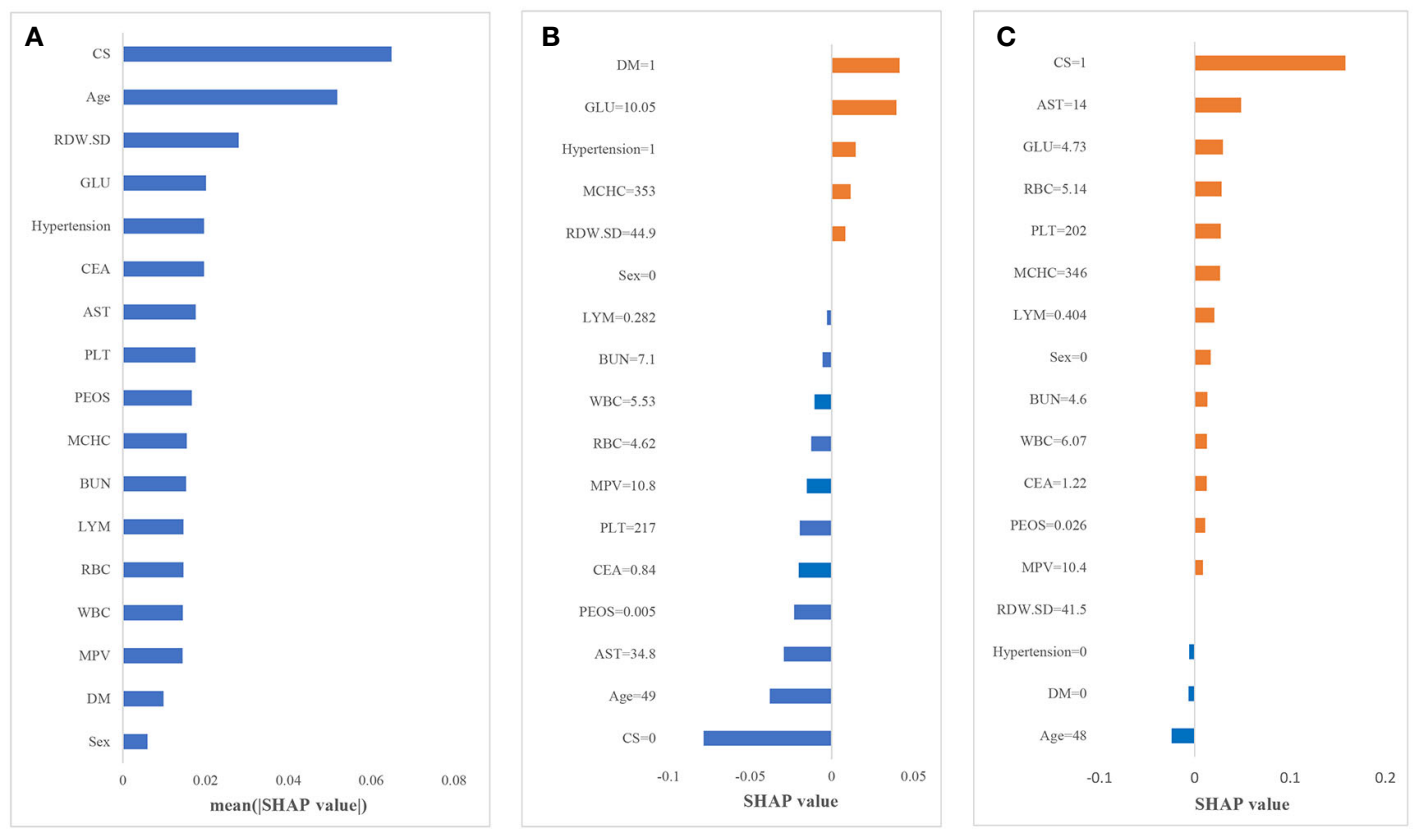
Results of the SHAP analysis. **(A)** Mean (|SHAP value|) of each variable; **(B)** the contribution of each variable in non-CAS individual I; **(C)** the contribution of each variable in CAS individual II.

In this paper, the SHAP value was used to quantify the impact of each variable on the prediction of CAS, and the results are shown in **Figure 3**. **Figure 3A** shows the contribution of all the features to the prediction, which was sorted according to the average SHAP values. CS and age are the two most important predictors with the largest SHAP values, followed by RDW-SD, GLU and hypertension.

To further explain how each variable affects the occurrence of CAS, we illustrate two sample cases. **Figures 3B, C** depict the SHAP value of each variable for individuals I and II. The blue bars on the left (SHAP value less than 0) indicate variables that reduce the probability of the individual being predicted as CAS; the orange bars on the right (SHAP value greater than 0) indicate variables that increase the probability of the individual being predicted as CAS. Larger areas indicate greater impacts of that factor. For individual I, diabetes and an increase in glucose are the main reasons for the increased risk of CAS. Due to the absence of CS, relatively young age and other variables with negative impacts, the predicted probability of CAS for individuals is low. In contrast, for individual II, CS is the main reason for the increased risk of CAS, and most of the variables have a positive impact in predicting CAS. The probability of CAS for individual II is only slightly reduced by the absence of hypertension, diabetes and young age; thus, this individual is more likely to develop CAS in the future. Therefore, through the SHAP framework, we can directly determine the main causes for the increased individual probability of CAS; thus, corresponding interventions could be taken to reduce the risk.

## 4 Discussion

In this study, based on a routine health check-up cohort, we constructed a stacking ensemble prediction model for quantifying the risks of incident CAS. Demographic information, such as age and sex, and clinical factors, including BUN, LYM, AST, RDW-SD, RBC, MCHC, MPV, GLU, PLT, PEOS, WBC, CEA, CS, hypertension and DM, were important predictors of CAS.

We established five machine learning models to predict CAS and found that the performance of the individual models varied in the testing and validation sets. Most of the models performed better on the testing set and inferiorly on the hold-out validation set, indicating the overfitting problem. Therefore, we used the super learner algorithm to integrate the models, which significantly improved their performance. The super learner model not only demonstrated superior discrimination but also effectively managed overfitting, with AUC scores of 0.893 and 0.861 in the testing and validation sets, respectively. Our findings align with recent research that demonstrates the superior performance of stacking models in various biomedical applications (31–33). Studies like those conducted by Zhou have shown that stacking models provide enhanced accuracy in predicting diabetes which is consistent with our results (34).

In accordance with several studies (4, 5, 35), age was identified as a risk factor for CAS. According to the results of the feature importance analysis for the three machine learning models and the SHAP explanatory framework, we found that age and CS were the two most important factors affecting the occurrence of CAS. The demographic shift towards an aging population warrants increased societal attention, given the anticipated rise in CAS incidence (36). Additionally, since CS is a symptom of CAS, its presence is an important signal of CAS, and these two groups of people in particular need to take corresponding measures to prevent CAS. Moreover, our analysis extends beyond conventional risk factors by incorporating endocrine-related markers within the predictive framework. The integration of these markers, including but not limited to abnormal blood glucose levels and hypertension, underscores the intricate relationship between endocrine dysfunctions and atherosclerosis. Through the SHAP framework, the contribution of each feature to the CAS risk is quantified, offering a personalized risk assessment. It underscores the necessity of a multifaceted risk assessment strategy that not only considers traditional factors like age and CS but also gives weight to the underlying endocrine dysfunctions contributing to the disease’s pathogenesis.

The SHAP score becomes an invaluable tool for clinicians, enhancing the interpretability of machine learning predictions and enabling personalized preventive measures. For instance, older individuals with CS may benefit from increased screening and early intervention strategies, facilitating early detection and management of CAS. Similarly, for individuals with abnormal blood glucose or hypertension, personalized medical interventions including adjustments in medication and lifestyle changes such as diet and exercise could be advised based on their specific risk profiles. Regular monitoring of blood pressure and glucose levels can further aid early intervention and management, demonstrating the dynamic utility of predictive models in clinical settings.

One of the limitations of our study was that information about important risk factors for CAS, such as lifestyle, was not available. However, the model in our study still achieved acceptable performance without these predictors. Moreover, study subjects in the routine check-up cohort were limited to a single source area, and the prediction model was only internally validated. External validation with an independent population is needed to evaluate the generalizability of the model. Future studies will aim to collaborate with various institutions across different geographic regions to ensure that our models are robust and applicable to a broader population. This approach will not only help in validating our current model externally but also in assessing its effectiveness across different demographic settings.

## 5 Conclusion

In this study, we developed a stacking model to predict the risk of incident CAS, enhancing the application of machine learning in the disease prediction. This approach not only provides a new method for the risk calculation of CAS but also highlight the critical role of endocrine dysfunctions in CAS development. By integrating a comprehensive analysis of predictors, and utilizing SHAP for model interpretation, our model effectively identifies high-risk individuals. This allows for targeted interventions that could substantially reduce the health and economic burdens associated with CAS. The study demonstrates the potential of advanced machine learning techniques to enhance preventive healthcare strategies.

## Data availability statement

The raw data supporting the conclusions of this article will be made available by the authors. Further inquiries can be directed to the corresponding author.

## Ethics statement

The studies involving humans were approved by the Ethics Committee of the First Affiliated Hospital of Shandong First Medical University, and informed consent was obtained from all the participants The studies were conducted in accordance with the local legislation and institutional requirements.

## Author contributions

XZ: Conceptualization, Formal analysis, Methodology, Writing – review & editing. CT: Formal analysis, Writing – original draft. SW: Writing – review & editing. WL: Writing – review & editing. WY: Writing – original draft. DW: Writing – original draft. QW: Writing – original draft. FT: Conceptualization, Data curation, Writing – review & editing.
