# Supplementary material for: A stacking ensemble model for predicting the occurrence of carotid atherosclerosis
Source: Front Endocrinol (Lausanne). 2024 Jul 23;15:1390352. doi: 10.3389/fendo.2024.1390352 (PMC11300245; doi:10.3389/fendo.2024.1390352)
Supplement: Supplementary file 1 [file DataSheet_1.pdf]

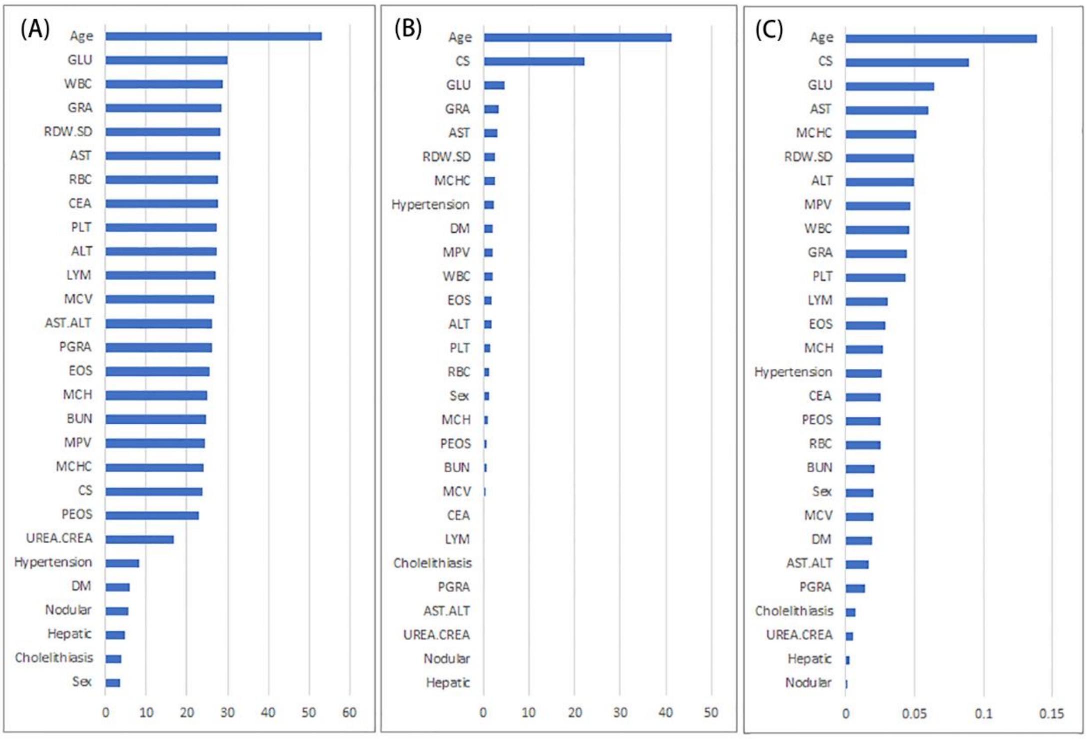

Figure S1 Variable importance for RF (A), GBDT (B), and XGBoost (C).

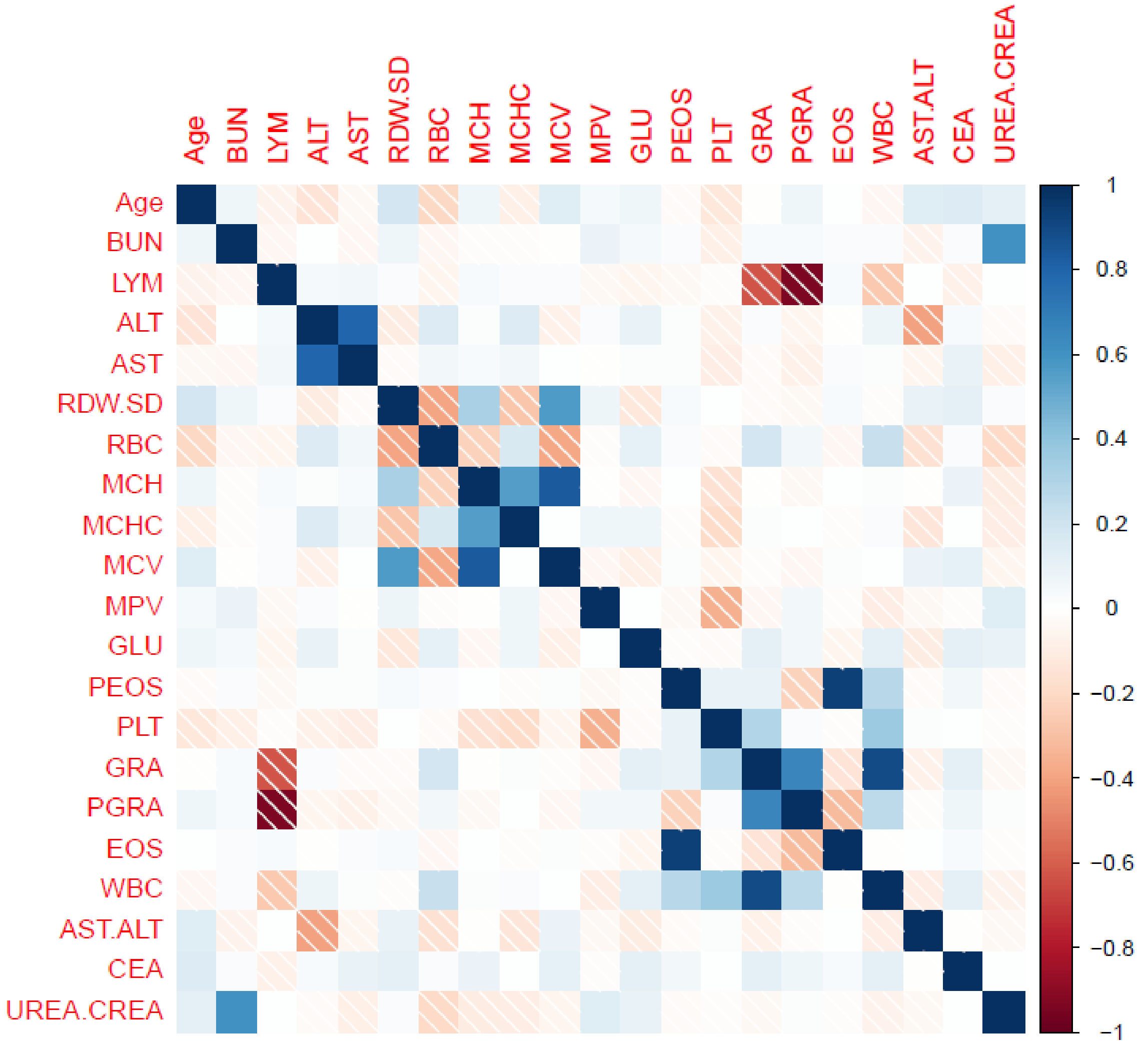

Figure S2 Correlation coefficient diagram of continuous features

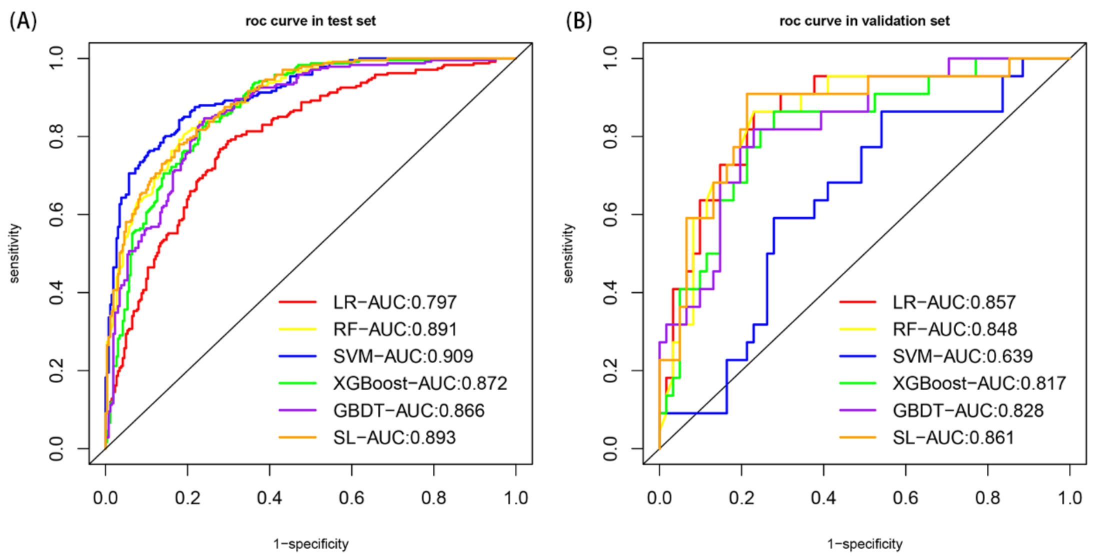

Figure S3. ROC curves for the six models in the testing set (A) and validation set (B). (LR, logistic regression; RF, random forest; SVM, support vector machine; XGBoost, extreme gradient boosting; GBDT, gradient boosting decision tree; SL, super learner)

Table S1 Weight coefficients of the super learner model

Model	weight coefficient
RF	0.820
LR	0.000
SVM	0.000
GBDT	0.089
XGBoost	0.091

Table S2 Hyperparameter used in the models

Hyperparameter	value
**RF**	
mtry	2
**SVM**	
kernel	radial
C	44.2
gamma	0.463
**XGBoost**	
eta	0.127
max_depth	5
min_child_weight	1.05
subsample	0.896
colsample_bytree	0.785
nrounds	300
**GBDT**	
shrinkage	0.324
interaction.depth	5
n.minobsinnode	7
bag.fraction	0.943
n.trees	300
distribution	bernoulli
